# Mesh-like electrospun membrane loaded with atorvastatin facilitates cutaneous wound healing by promoting the paracrine function of mesenchymal stem cells

**DOI:** 10.1186/s13287-022-02865-5

**Published:** 2022-05-07

**Authors:** Jieyu Xiang, Ling Zhou, Yuanlong Xie, Yufan Zhu, Lingfei Xiao, Yan Chen, Wei Zhou, Danyang Chen, Min Wang, Lin Cai, Liang Guo

**Affiliations:** 1 Department of Plastic Surgery, Zhongnan Hospital of Wuhan University, Wuhan, 430071 China; 2 Department of Spine Surgery and Musculoskeletal Tumor, Zhongnan Hospital of Wuhan University, Wuhan, 430071 China

**Keywords:** Mesh-like topography, Electrospun fibrous, Atorvastatin, Bone marrow stem cells, Paracrine secretion, Tissue engineering, Wound healing

## Abstract

**Background:**

Functional electrospun membranes are promising dressings for promoting wound healing. However, their microstructure and drug loading capacity need further improvements. It is the first time to design a novel mesh-like electrospun fiber loaded with atorvastatin (ATV) and investigated its effects on paracrine secretion by bone marrow-derived mesenchymal stem cells (BMSCs) and wound healing in vivo.

**Methods:**

We fabricated a mesh-like electrospun membrane using a copper mesh receiver. The physical properties of the membranes were evaluated by SEM, FTIR spectroscopy, tensile strength analysis, and contrast angle test. Drug release was measured by plotting concentration as a function of time. We tested the effects of conditioned media (CM) derived from BMSCs on endothelial cell migration and angiogenesis. We used these BMSCs and performed RT-PCR and ELISA to evaluate the expressions of vascular endothelial growth factor (VEGF) and basic fibroblast growth factor (b-FGF) genes and proteins, respectively. The involvement of FAK and AKT mechanotransduction pathways in the regulation of BMSC secretion by material surface topography was also investigated. Furthermore, we established a rat model of wound healing, applied ATV-loaded mesh-like membranes (PCL/MAT) seeded with BMSCs on wounds, and assessed their efficacy for promoting wound healing.

**Results:**

FTIR spectroscopy revealed successful ATV loading in PCL/MAT. Compared with random electrospun fibers (PCL/R) and mesh-like electrospun fibers without drug load (PCL/M), PCL/MAT induced maximum promotion of human umbilical vein endothelial cell (HUVEC) migration. In the PCL/MAT group, the cell sheet scratches were nearly closed after 24 h. However, the cell sheet scratches remained open in other treatments at the same time point. The PCL/MAT promoted angiogenesis and led to the generation of longer tubes than the other treatments. Finally, the PCL/MAT induced maximum gene expression and protein secretion of VEGF and b-FGF. As for material surface topography effect on BMSCs, FAK and AKT signaling pathways were shown to participate in the modulation of MSC morphology and its paracrine function. In vivo, PCL/MAT seeded with BMSCs significantly accelerated healing and improved neovascularization and collagen reconstruction in the wound area compared to the other treatments.

**Conclusions:**

The mesh-like topography of fibrous scaffolds combined with ATV release creates a unique microenvironment that promotes paracrine secretion of BMSCs, thereby accelerating wound healing. Hence, drug-loaded mesh-like electrospun membranes may be highly efficacious for wound healing and as artificial skin. It is a promising approach to solve the traumatic skin defect and accelerate recovery, which is essential to developing functional materials for future regenerative medicine.

**Supplementary Information:**

The online version contains supplementary material available at 10.1186/s13287-022-02865-5.

## Background

The skin is the largest organ of the human body. It plays significant roles in sensory perception, absorption, secretion, and protection against the external environment, making it vital to human health [1]. The integrity of the skin can be disrupted by various chemical, mechanical, and thermal factors. Skin lesions jeopardize the health of the underlying tissues [2] and pose risks to the entire body by permitting access to microbial pathogens [3]. The use of autologous grafts to cover defects with insufficient blood supply is constrained by donor skin shortage [4]. Ideal wound dressings are biocompatible, soft, conserve moisture within the wound, isolate the wound from the external environment, avoid secondary damage, and enhance cell growth under the wound [5, 6]. Without the assistance of suitable dressings, natural tissue injury repair may be unsatisfactory and skin regeneration may be poor. Hence, clinical skin repair may be ineffective in the absence of appropriate wound dressings.

Tissue engineering combines cells, biocompatible extracellular scaffold materials, and appropriate biochemical factors to facilitate repair and replacement of damaged tissues [7]. Mesenchymal stem cells (MSCs) have been extensively studied in regenerative medicine. They have attracted increasing attention among researchers due to their safety and efficacy profile for tissue regeneration [8]. The paracrine secretions from MSCs induce various effects including angiogenesis, immunoregulation, anti-scarring, anti-apoptosis, chemotaxis, and local cellular regulation [9, 10]. MSCs secrete proangiogenic vascular endothelial growth factor (VEGF) and basic fibroblast growth factor (b-FGF) that coordinate multiple biological processes required for tissue regeneration. Thus, VEGF and b-FGF are vital for efficient wound healing [11, 12].

Scaffolds are key tissue engineering components as they serve as substrates upon which cells attach and proliferate [13]. Scaffold with suitable structures, surface topography, and chemical characteristics create appropriate environments for cell growth and affect cellular secretory functions via cell–material interactions [14].

Atorvastatin (ATV) is a 3-hydroxy-3-methylglutaryl coenzyme A (HMG-CoA) reductase inhibitor that lowers blood lipid levels, has a long half-life, yields active metabolites, and has a high protein binding rate [15]. In rats, topical ATV treatment promotes tissue regeneration in acute lesions and regulates the expression of cell growth-related proteins and cytokines [16, 17]. ATV promotes wound healing and exerts anti-inflammatory and proangiogenic effects [16]. Topical application of ATV has demonstrated excellent long-term efficacy without causing the adverse effects associated with oral ATV administration [17]. Exosomes derived from bone marrow MSCs (BMSCs) pretreated with ATV exhibit superior proangiogenic effects in diabetic wound healing [18]. Moreover, ATV protects MSCs from hypoxia and serum deprivation-induced damage and strengthens their paracrine effect [19–21].

EFs offer numerous advantages such as convenience of fabrication and have become important scaffold materials in tissue engineering [22–24]. In the present study, we selected electrospun fibers (EFs) to investigate the effects of mesh-like topography and of ATV loading on these EFs on MSC paracrine function. It is the first time to evaluate the impact of a combination of mesh-like topography and atorvastatin loading on the paracrine function of MSCs.

**Fig. 1:**
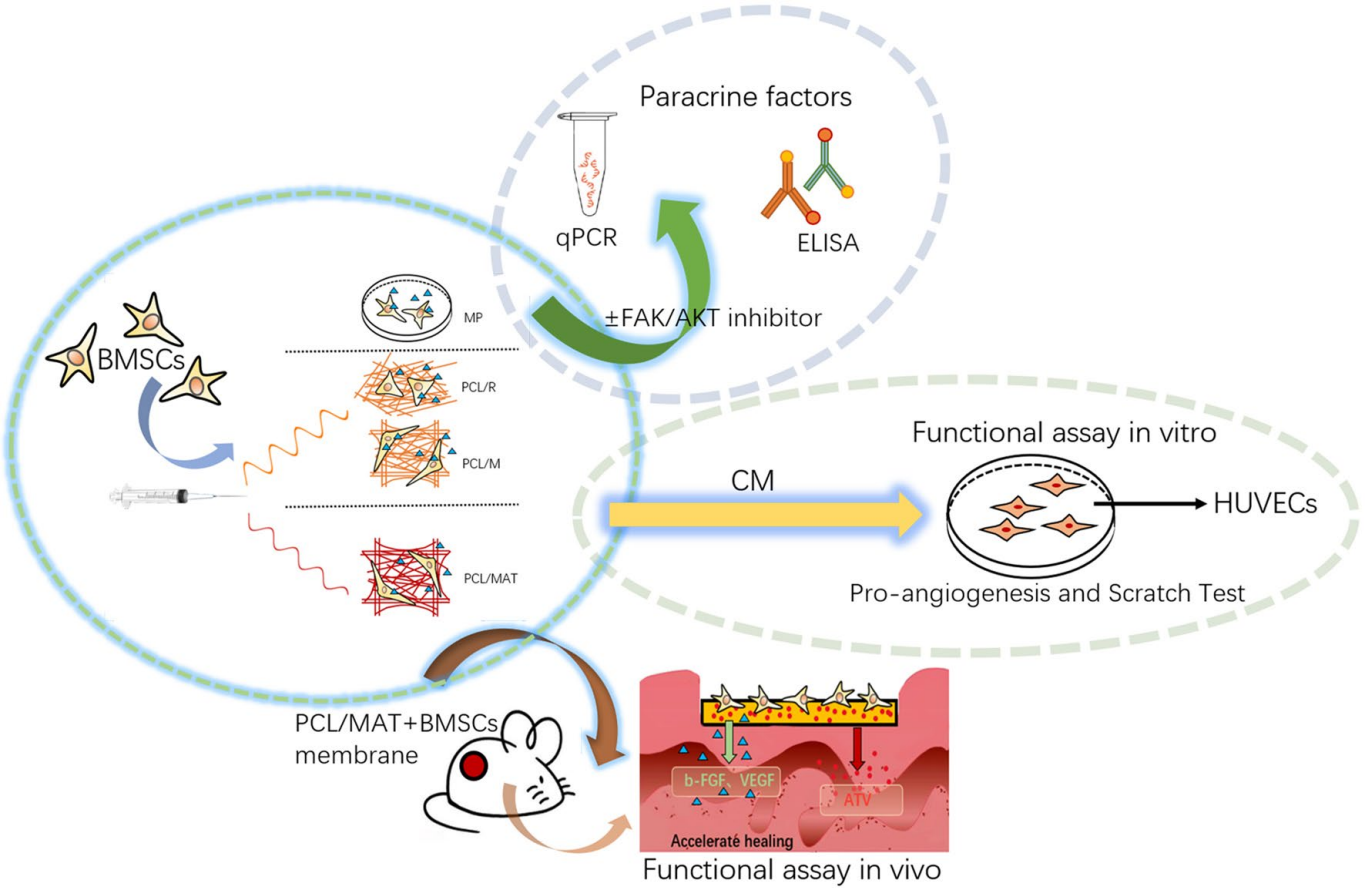
The experimental designed to investigate the influence of the fiber morphology and atorvastatin (ATV) loaded in the fiber on the paracrine secretion function of BMSCs. The scaffolds for cell culture included electrospun fibers (EFs) in random, a mesh-like organization, and the mesh-like organization with atorvastatin loaded in, which were designated as PCL/R, PCL/M and PCL/MAT, and the cultures on EFs were compared to the BMSCs cultured on polystyrene microplate (MP). Then the PCL/MAT membranes seeded with BMSCs were used as the artificial skin in vivo wound healing

Figure 1 shows polycaprolactone (PCL) EFs with random and mesh-like morphology (PCL/R and PCL/M, respectively) and ATV-loaded mesh-like membrane (PCL/MAT). Later, we investigated their effects on the ability of rat BMSCs to produce paracrine proangiogenic factors. We examined the functions of the paracrine factors by collecting conditioned media (CM) from various culture systems and applying them to endothelial cell cultures. Figure 1 shows that BMSCs were seeded onto a PCL/MAT scaffold to functionalize it as an artificial skin. We assessed the capacity of this biomaterial to promote wound healing in vivo. Our results suggest that mesh-like topography combined with ATV loading dramatically enhanced the paracrine function of the BMSCs. Thus, PCL/MAT scaffold seeded with BMSCs may be an excellent skin tissue reconstruction biomaterial to promote skin wound healing.

## Methods

### Cell culture

BMSCs were isolated from the femurs of juvenile Sprague Dawley rats aged 4 weeks. The ends of the femurs were cut off with scissors and placed in culture medium, and the BMSCs were isolated from the bone marrow by density gradient centrifugation. The BMSCs were seeded in a T25 culture flask and cultured in alpha minimum essential medium (α-MEM) containing 10% (v/v) fetal bovine serum (FBS; Gibco, Grand Island, NY, USA) and 1% (v/v) penicillin–streptomycin at 37 °C in a humidified CO_2_ incubator. The culture medium was changed every 3 days, and phosphate-buffered saline (PBS) was used to remove unattached BMSCs. The BMSCs were passaged when they reached 80% confluence. Only early passages (p3–p5) were used.

Human umbilical vein endothelial cells (HUVECs; ATCC No. CRL-1730) were procured from the BeNa Culture Collection (Beijing, China) and cultured in endothelial cell medium (ECM) supplemented with 10% (v/v) FBS and 1% (v/v) penicillin–streptomycin at 37 °C in a humidified CO_2_ incubator. The medium was replaced every 3 days, and cells were cultured until they reached 80% confluence. The BMSCs were cultured in FBS-free MEM-α for a prescribed period to collect CM. For propagation and experiments, BMSCs and HUVECs were detached from their culture plates using trypsin/EDTA.

### Fibrous scaffold fabrication

EF scaffolds were prepared from polycaprolactone (PCL) solution. Briefly, 10% (w/v) PCL was dissolved in 9:1 (v/v) chloroform/dimethylformamide. The suspension was stirred for 2 h until the PCL was fully dissolved. To prepare ATV-loaded EF (PCL/MAT), different amounts of ATV powder (2.5, 25, and 250 mg) were added to the PCL solution and the mixture was stirred for 1 h. Then, the syringe was filled with 5 mL solution. The blunt end needle is stainless 21-gauge. The infusion speed and applied voltage were fixed at 0.5 mL/h and 12 kV, respectively. The collection distance was 13 cm for both the random EF (PCL/R) and the mesh-like EF (PCL/M and PCL/MAT). The PCL/R was collected on a flat plate, whereas the PCL/M and PCL/MAT were collected on a copper mesh. The EF scaffolds were sterilized in a biosafety cabinet UV irradiation, 2 h per side, and stored in sterile dishes at 4 °C until use.

### Scanning electron microscopy (SEM) images

Topographical features of the scaffolds were examined by SEM (VEGA 3 LMU; TESCAN, Czech Republic). Mean scaffold diameters were calculated from the SEM images using ImageJ.

### Fourier transform infrared (FTIR) spectroscopy

To confirm successful ATV loading in the EFs, PCL/M, PCL/MAT, and raw ATV powder samples were analyzed by FTIR spectroscopy (Nicolet 5700; Thermo Fisher Scientific, USA). The IR spectra were measured in the 4000–400 cm^−1^ wavelength range.

### Tensile strength analysis

The uniaxial tensile strengths of the electrospun scaffolds were evaluated using a tensile testing machine (CMT6503, Shenzhen SANS Test Machine, China). The three types of fibrous membranes were cut into rectangles (40 mm × 10 mm), fixed on the molds, and stretched at a constant rate of 5 mm/min until breakage. The tensile strengths and Young’s moduli were interpolated from stress–strain curves.

### Hydration behavior

Contact angle tests were conducted at room temperature using a contact angle goniometer (GBX, Digidrop, France). At least two measurements were taken at two points per scaffold specimen. The contact angle was measured after 10 s.

### Drug release test

To detect ATV release from the samples, various concentrations of drugs were added to 50% (v/v) methanol–PBS solutions and a concentration curve was plotted. The ATV-loaded electrospun membranes (PCL/MAT-ATV = 2.5 mg) were cut into squares, with an average weight of 10 mg each. Then, each sample was immersed in 10 mL PBS (pH 7.4) at 37 °C. At predetermined time points, 2 mL release medium was added to 2 mL methanol and the mixture was subjected to ultrafiltration before detection. An equal volume of fresh PBS was added to replace the release medium drawn from release system. The amount of released ATV in the mixture was measured using a microplate reader (Thermo Fisher Scientific) at 246 nm [25].

### Cell proliferation assay

Cell proliferation was investigated using a Cell Counting Kit (CCK)-8 assay at different time points. The BMSCs were seeded on the surfaces of membranes (PCL/R, PCL/M, PCL/MAT—2.5 mg\25 mg\250 mg) placed in wells (1 × 10^4^/well) and incubated for 1, 3, or 5 days. The cells were rinsed twice with PBS; then, 30 μL CCK-8 solution and fresh culture medium (300 μL) were added to each well, followed by 2-h incubation at 37 °C. After that, 100 μL supernatant was transferred to a 96-well plate and the absorbance was measured at 450 nm using a microplate reader (Thermo Fisher Scientific). Cell proliferation was determined using the absorbance of the different treatment groups.

### Fluorescent confocal imaging of cell adhesion

Scaffolds placed in wells were seeded with BMSCs (1 × 10^4^/well) and fixed overnight in 2% (v/v) paraformaldehyde (PFA). The cells were stained with rhodamine phalloidin for 1 h and with DAPI for 5 min. The stained adherent cells were imaged by CLSM (TCS SP8, Leica).

### RT-PCR assay

The *VEGF* and *b-FGF* expression levels in the BMSCs were measured by performing RT-PCR. The BMSCs were seeded (1 × 10^4^/mL) and incubated on the PCL/R, PCL/M, PCL/MAT, and MP surfaces for 3 days. The cells were washed twice with PBS, and total RNA was harvested from the cells using TRIzol reagent. The RNA concentrations were determined by spectrophotometry. Reverse transcription was performed using a HiScript III RT SuperMix reverse transcription kit for quantitative polymerase chain reaction (qPCR) following the manufacturer’s protocol. After reverse transcription, real-time PCR was performed using a 7500 Real-Time PCR system.

(Applied Biosystems, USA) with ChamQ SYBR qPCR Master Mix. The expression of each gene was normalized to that of GAPDH. The following primer sequences were used for amplification: GAPDH (Forward, TGAAGGTCGGAGTCAACGGATTTG; Reverse, CATGTGGGCCATGAGGTCCACCAC); VEGF (Forward, TTCATGGATGTCTATCAGCG; Reverse, GCTCATCTCTCCTATGTGCT); b-FGF (Forward, GTCAAACTACAGCTCCAAGCAGAA; Reverse, AGGTACCGGTTCGCACACA).

### VEGF-A and b-FGF secretion

The concentrations of VEGF and b-FGF secreted in the culture media were measured using an ELISA kit according to the manufacturer’s instructions. The membrane surfaces (PCL/R, PCL/M, PCL/MAT) and the control (MP) were inoculated with BMSCs (2 × 10^4^/mL) and incubated for 3 days. The supernatants were collected by 30-min centrifugation at 10,000 rpm, and their OD at 450 nm was measured using a microplate reader (Thermo Fisher Scientific). The VEGF-A and b-FGF concentrations were determined from a standard curve. Each experiment was conducted in triplicate.

### Cell migration assay

The chemotactic effects of various CMs on HUVEC migration were determined by performing an in vitro cell migration assay. Briefly, HUVECs (1 × 10^5^/well) were seeded in six wells, cultured in half-fresh complete medium or half-conditioned medium (PCL/R-CM, PCL/M-CM, PCL/MAT-CM, and MP-CM), and incubated until they reached ~ 90% confluence. The cell monolayers were scratched with a sterile pipette tip to form wounds. HUVECs cultured in normal medium served as the control. After 24-h incubation, the samples were observed under bright-field microscopy and cell motility was quantitated by observing the widths of the gaps in the healing wounds.

### Endothelial tube formation assay

Briefly, HUVECs (1 × 10^5^ cells/well) were seeded onto Matrigel films placed in 24-well plates and treated with 50% CM or 50% ECM supplemented with 2% (v/v) FBS. The HUVECs were incubated for 6 h and imaged under bright-field microscopy. Tube lengths were measured using AngioTool 0.6 software.

### Western blot analysis

The protein expression levels were detected by WB on MSCs cultured on the mesh-like and random scaffolds after 3 days of culture. The total protein was extracted, separated through sodium dodecyl sulfate–polyacrylamide gel electrophoresis (SDS-PAGE), and transferred to polyvinylidene fluoride membranes, which were incubated with primary antibodies against FAK (#3285, CST, USA), phosphorylated-FAK (#3283, CST, USA), AKT (#9272, CST, USA), and p-AKT (#9271, CST, USA) overnight at 4 °C. After washing, the membranes were incubated with horseradish peroxidase-conjugated secondary antibodies for 1 h at room temperature. The gray value of GAPDH was used for normalization of total protein. Densitometric analysis of the Western blot bands was then conducted using ImageJ software.

### Pathway inhibition assay

The involvement of FAK and AKT in regulating the mesh-like topography-mediated MSCs paracrine function was verified using inhibitors of FAK (PF573228, Sigma-Aldrich, USA, 10 μM) and AKT (LY294002, Selleck, China, 50 μM). The protein expression levels of FAK, p-FAK, AKT, and p-AKT in MSCs cultured on the mesh-like scaffolds with FAK and AKT inhibitor co-incubation were detected using WB analysis, while mRNA and its secretory protein of VEGF and b-FGF in MSCs cultured on the mesh-like scaffolds with or without inhibitor addition were measured via RT-PCR and ELISA.

### Animal model of wound healing

A rat model of wound healing was established to evaluate and compare wound healing efficiency of different membrane treatments. Briefly, 24 male Sprague Dawley rats (180–220 g) were used for wound induction. The animals were anesthetized by forced 5% isoflurane inhalation, their dorsal fur was shaved, and target areas were sterilized with povidone-iodine. Two round excisional skin wounds of 10 mm full thickness were created on each side of the midline using a biopsy punch. A donut-shaped silicone splint was glued to the skin with biomedical glue, and the wound was centered in the splint. The latter stabilized the wound and prevented it from contracting. The rats were randomly assigned to the various treatment groups. For the blank control, no membrane was placed around the wound. In different treatment groups (PCL/MAT + BMSCs, PCL/M + BMSCs, PCL/R + BMSCs, PCL/MAT), round 10-mm membrane pieces were wrapped around wound. BMSCs (1 × 10^4^ cells per cm^2^) were seeded onto each PCL membrane. The negative and positive controls had gauze and commercial chitosan dressings, respectively. Wound images were recorded using a DSLR camera (Canon Inc., Tokyo, Japan) at 0, 7, and 14 days after surgery. The rats were killed on days 7 and 14 after surgery.

### Histological analysis and immunostaining

The rats were euthanized, and the healthy tissues surrounding their wounds were excised and fixed in 4% (v/v) paraformaldehyde overnight. The samples were dehydrated using a graded series of ethanol solutions (70%, 85%, 90%, 95%, and 100%) and xylene (50% and 100%), embedded in paraffin, and sectioned to 7 μm thickness using a Leica RM 2245 microtome (Leica Microsystems, Wetzlar, Germany). All samples were subjected to H&E, Masson’s trichrome (Sigma-Aldrich Corp., St. Louis, MO, USA), CD31 and CD206 immunostaining. The samples were visualized under inverted and upright fluorescence microscopes. New blood vessels were enumerated using the ImageJ software (NIH).

### Statistical analysis

All data are presented as means ± standard error of the mean (SEM). One-way ANOVA was performed to evaluate significant differences between treatments. Results with *P* < 0.05 were considered statistically significant. GraphPad Prism v. 6 (GraphPad Software, La Jolla, CA, USA) was used for the statistical analyses.

## Results

### Electrospun fibrous matrix preparation and BMSC co-culture

To investigate the effects of scaffolds on paracrine secretion from BMSCs, we fabricated three types of electrospun membranes, cultured BMSCs with them, and used CMs to culture HUVECs. All three EFs showed random, mesh-like, and ATV-loaded mesh-like patterns designated PCL/R, PCL/M, and PCL/MAT, respectively. The BMSCs were seeded onto the membranes, and expressions of VEGF and b-FGF encoding genes and proteins were analyzed using RT-PCR and ELISA, respectively. The functions of the paracrine molecules derived from different culture systems were investigated by collecting the CMs and applying them to the HUVEC cultures. In the in vitro experiments, cells cultured on polystyrene microplates (MP) were used as controls to understand how materials with fibrous topology affect paracrine secretion in BMSCs. The functions of the PCL/MAT membrane seeded with BMSCs as artificial skin were investigated in an in vivo model of wound healing.

### Membrane morphology and characterization

**Fig. 2:**
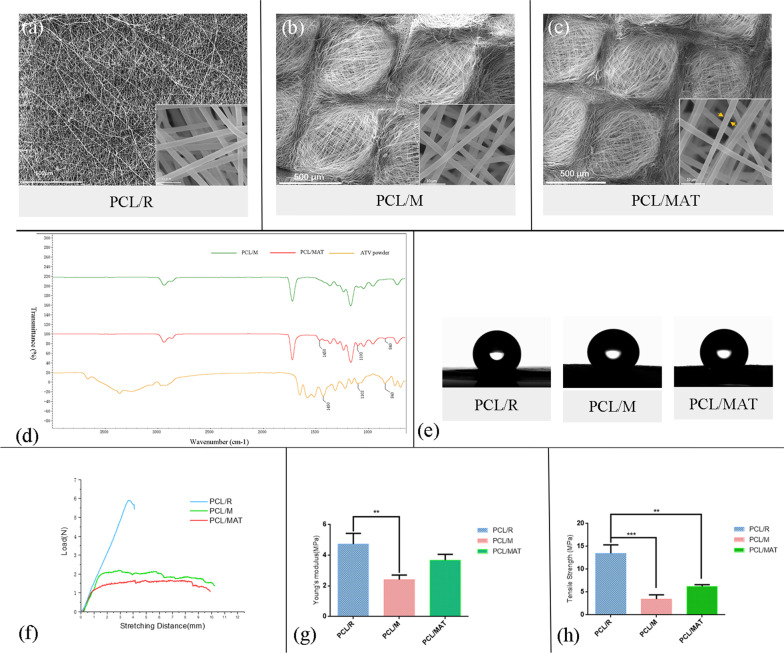
**a**–**c** SEM images of PCL/R, PCL/M, and PCL/MAT, respectively. SEM micrographs of electrospun fibers at a magnification of 100X (scale bar = 500 µm) and a magnification of 5000X (scale bar = 10 µm). **d** FTIR spectra of ATV powder, PCL/M and PCL/MAT; **e** Water droplet in contact with PCL/R, PCL/M, and PCL/MAT. **f** The stress–strain curves, **g** Young's modulus and **h** tensile strength. **Indicates significant difference of *p* < 0.01. ***Indicates significant difference of *p* < 0.001

We fabricated scaffolds with distinct morphologies using various collectors in the electrospinning process. We then investigated the physicochemical properties of these membranes. The PCL/R contained random fibers, whereas the PCL/M and PCL/MAT collected with copper mesh consisted of vertically oriented bundled fibers in lattice patterns, with a grid length of 650 μm (Fig. 2a–c). The diameters of the PCL/R, PCL/M, and PCL/MAT fibers were 1.854 ± 0.047 μm, 1.876 ± 0.023 μm, and 1.782 ± 0.055 μm, respectively. The EFs of each membrane were round, continuous, and nearly bead-free. The spinning surface of the ATV-loaded group was more uneven than that of the unloaded group (orange arrow; Fig. 2). FTIR spectra are shown for pure ATV raw powder, pure PCL fibers (PCL/M), and ATV-loaded-PCL fibers (PCL/MAT) (Fig. 2d). For the pure ATV drug, characteristic peaks were observed at 1450 cm^−1^(C=C) stretching, 840 cm^−1^ (C−H) stretching, and 1103 cm^−1^ (C−F) stretching. These were related to the phenyl groups present in ATV. Similar peaks were observed for the PCL/MAT fiber, but not for PCL/M fiber. Hence, ATV was successfully loaded into PCL/MAT.

We also investigated the hydrophilicity and mechanical strength of the prepared membranes. The water contact angle of the random fibers (PCL/R) was 129.29° ± 0.60, while angles for the mesh-like scaffolds with and without ATV were 126.81° ± 2.65 and 132.27° ± 0.75, respectively (Fig. 2e). Statistical analysis indicated no significant difference between each group of membranes (*p* > 0.05). For the mechanical tests, the Young’s moduli and tensile stress of the EFs were calculated from stress–strain curves (Fig. 2f–h). The random membrane showed higher Young’s modulus and tensile stress than the mesh-like membrane.

### In vitro* drug release*

**Fig. 3:**
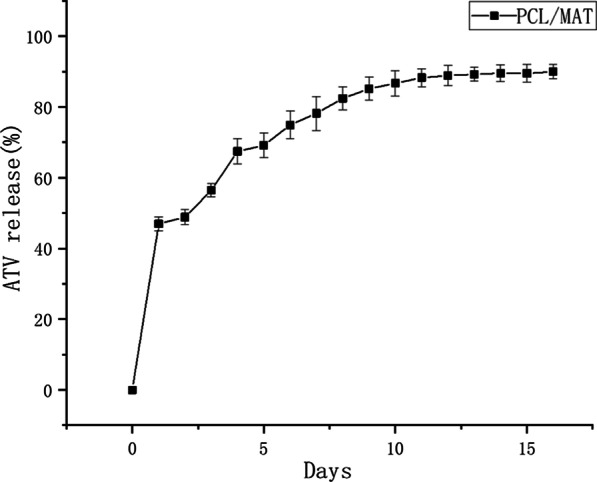
Release of atorvastatin from PCL/MAT electrospun fibers after incubation in PBS at 37 °C

Next, we investigated ATV release from the PCL/MAT membrane. Based on a concentration standard curve for ATV, we determined the cumulative concentrations released from PCL/MAT (Fig. 3). The release rate of ATV from the PCL/MAT membrane was ~ 43.69 ± 2.13% on the first day. By the fourth day, > 65.47 ± 1.96% of the ATV was released from the membrane. Over the next 5–7 days, the ATV was released relatively slowly and smoothly by the fibers. The platform release rate of the drug-loaded fiber membrane was 89.34 ± 2.16%.

### Biocompatibility of electrospun fibrous membranes

**Fig. 4:**
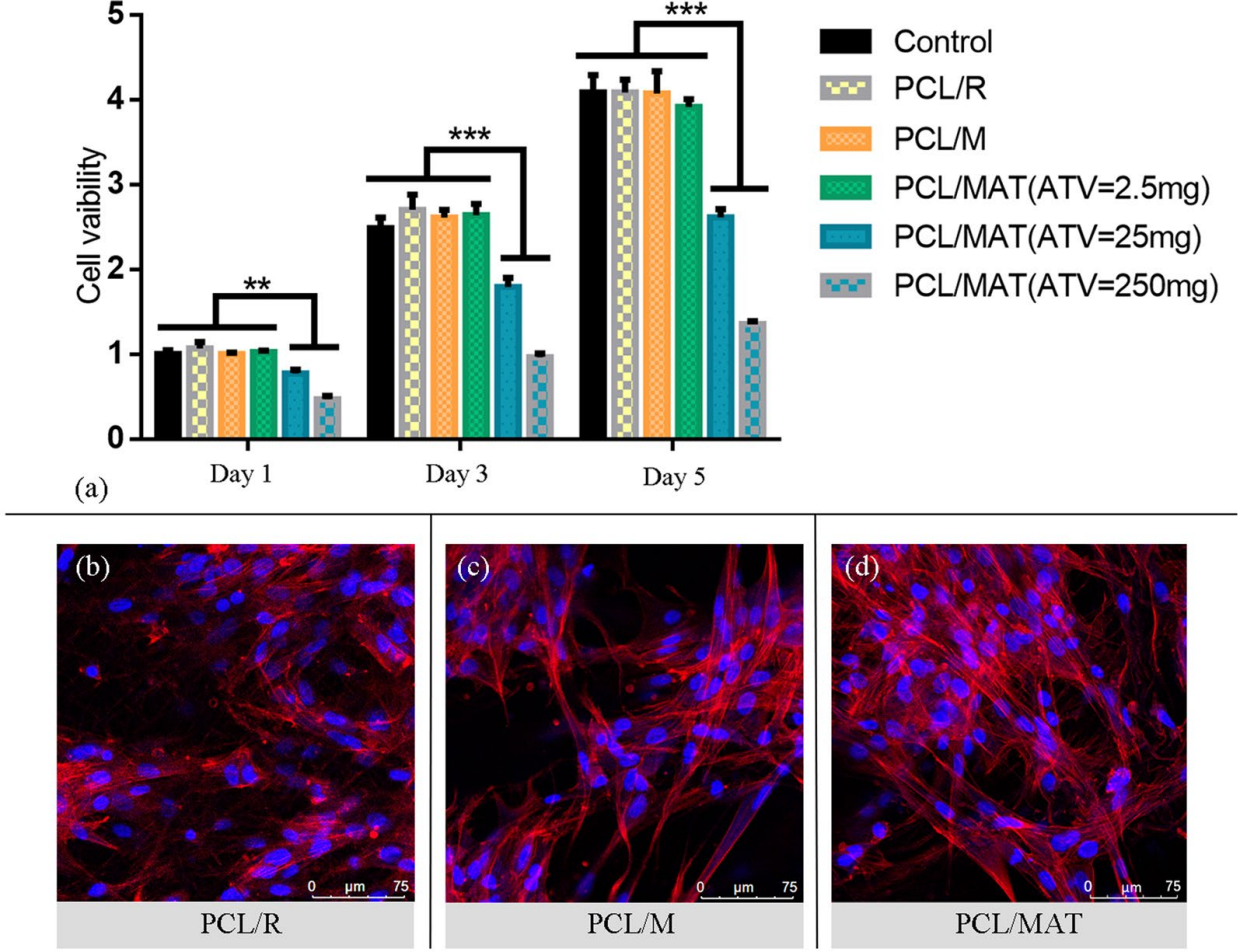
**a** Cell proliferation examined by a CCK-8 assay after BMSCs were cultured on different membranes for 1, 3, and 5 days. **b**–**d** Fluorescence microscopy images of BMSCs cultured on the surfaces of different samples PCL/R, PCL/M, and PCL/MAT for 3 days, respectively. (Red: cytoskeleton stained with rhodamine phalloidin; blue: nuclei stained with DAPI). **p < 0.01. ***p < 0.001

To characterize scaffold biocompatibility, BMSCs were seeded (1 × 10^4^/well) and cultured on various scaffolds using MP as the control. PCL/R, PCL/M, and PCL/MAT (ATV = 2.5 mg) were nontoxic to the BMSCs, whereas PCL/MAT (ATV = 25 mg) and PCL/MAT (ATV = 250 mg) inhibited BMSC growth (Fig. 4a). The optimal ATV/PCL mass ratio was 1:200 (2.5 mg:500 mg). Therefore, the PCL/MAT samples were produced using this mass ratio.

Fluorescence microscopy images (Fig. 4b–d) showed that the BMSCs adhered and grew on PCL/R, PCL/M, and PCL/MAT. Confocal microscopy revealed that the BMSCs on PCL/M and PCL/MAT were elongated and formed new membrane cytoskeleton as evident by the appearance of pseudopods. By contrast, the BMSCs on random fibers (PCL/R) were more circular than those on the mesh-like fibers (PCL/M, PCL/MAT).

### Migration and angiogenesis assays

**Fig. 5:**
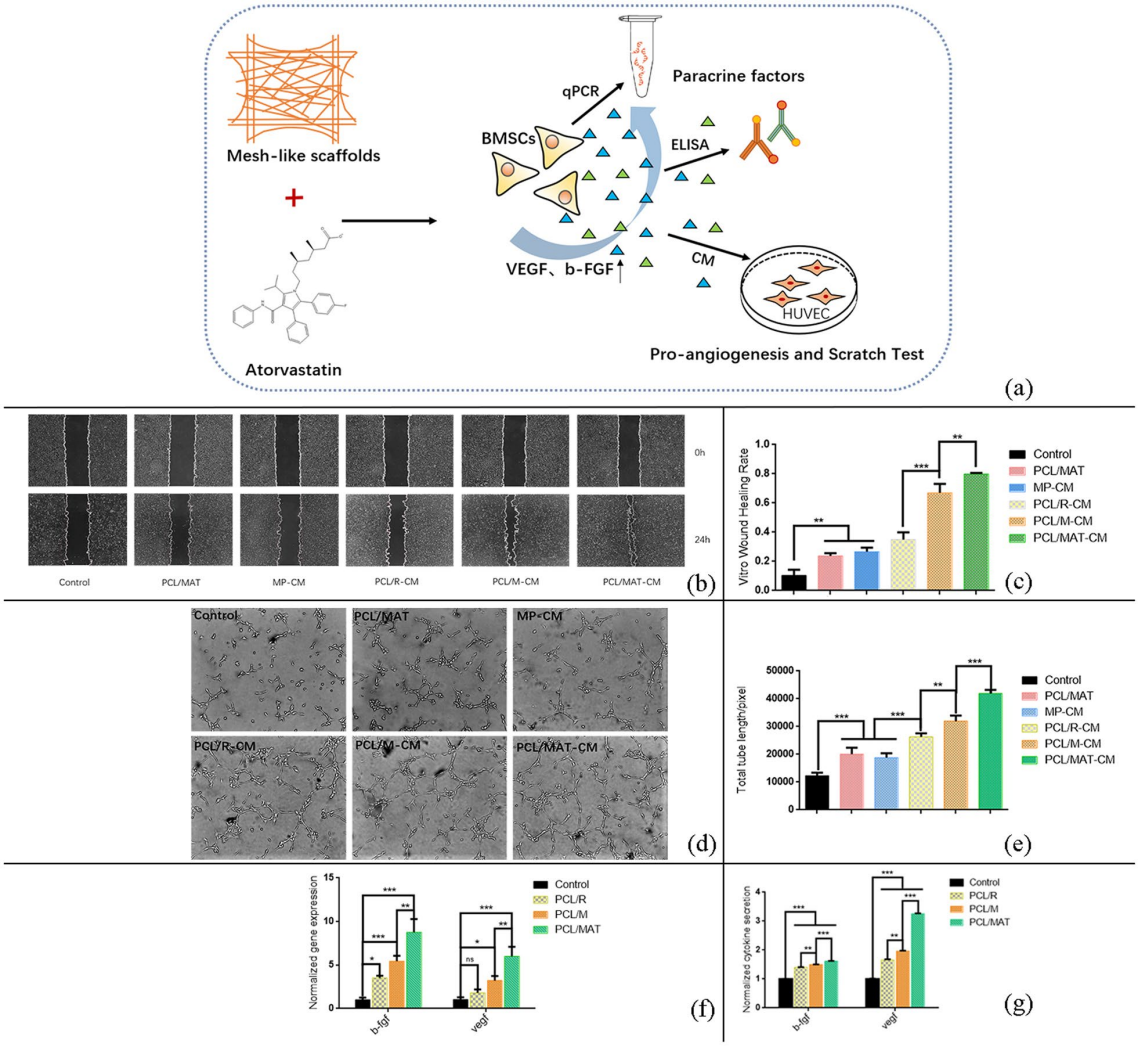
**a** Schematic diagram illustrated the effect of PCL/MAT scaffolds on the paracrine action of BMSCs and the experiment above. **b** The effects of the conditioned mediums (CMs) derived from BMSCs cultures on the HUVECs migration for up to 24 h and (d) HUVECs tube formation, **c** quantified results of **b**, **e** quantified results of the total tube length of **d**. **f**, **g** gene expression levels and secretion of b-fgf, vegf for different groups. *p < 0.05, **p < 0.01, ***p < 0.001

To clarify the functions of the molecules secreted from BMSCs, we collected conditioned serum-free media from BMSC cultures grown on different scaffolds and microplates and applied them to endothelial cell cultures (Fig. 5a). For the migration and angiogenesis assays, we used the supernatants derived from 3D BMSC cultures grown on MP, PCL/R, PCL/M, and PCL/MAT. The CMs from the EF scaffolds PCL/R-CM, PCL/M-CM, and PCL/MAT-CM promoted HUVEC migration more strongly than those from the microplate group (MP-CM) (Fig. 5b). Specifically, the CMs from the PCL/MAT were strongly inductive; moreover, the CMs from the mesh-like groups accelerated cell migration compared with the random group (PCL/M-CM vs. PCL/R-CM). Furthermore, the induction was significantly enhanced after ATV addition (PCL/MAT-CM vs. PCL/M-CM).

Analysis of the proangiogenic function of the CMs revealed that the total length of the vessel-like tubes formed by the cells in the four CM sample groups was greater than that of the control group (Fig. 5d), suggesting that the CMs had a trophic effect. Of the four different CM samples, the effects of those collected from the EF scaffolds (PCL/R-CM, PCL/M-CM, and PCL/MAT-CM) were significantly more potent than that of MP-CM. Moreover, the mesh-like scaffolds loaded with the ATV were most effective in facilitating the production of proangiogenic factors from the BMSCs. To exclude the influence of possible ATV release from the scaffolds on the HUVECs, we used the leachate solution from the ATV-loaded membranes to culture HUVECs in the migration and angiogenic-related assays. ATV released from the scaffolds slightly accelerated HUVEC migration and increased the total length of the vessel-like tubes (Fig. 5b–e).

The gene expression level and secretion of b-FGF and VEGF was notably higher in cells grown on all three membrane surfaces than in cells grown on MP, and maximum was observed in cells grown on the PCL/MAT fiber surface (Fig. 5f–g). In addition, the mesh-like group promoted the gene expression and protein secretion of b-FGF and VEGF more than the random groups (PCL/R vs. PCL/M). Furthermore, the ATV-loaded group promoted b-FGF and VEGF gene expression and protein secretion more than the no-drug group (PCL/MAT vs. PCL/M).

### Activation of FAK signaling pathway in BMSCs by mesh-like scaffolds

**Fig. 6:**
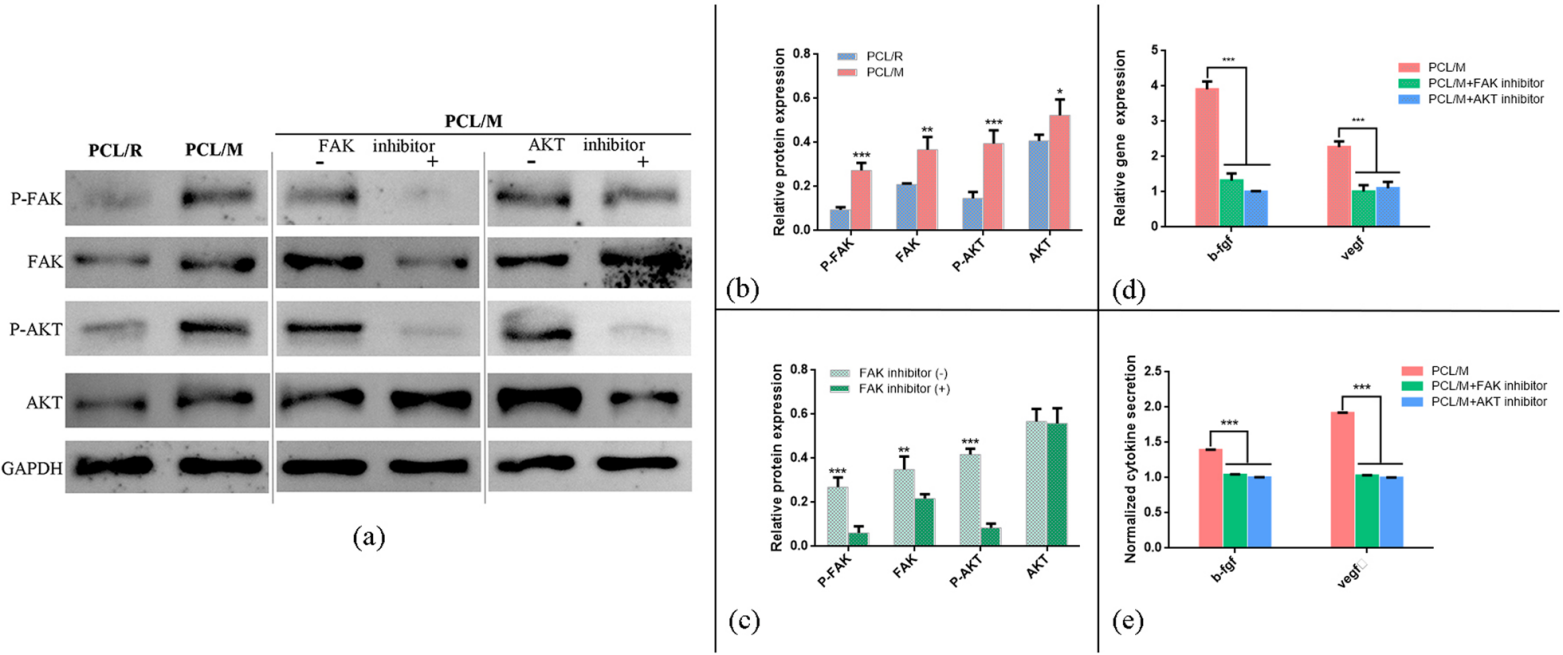
**a** Representative Western blot images and semiquantitative analysis of FAK and AKT signaling pathway protein expression **b** in MSCs cultured on the two kinds of scaffolds and **c** in MSCs cultured on the mesh-like scaffold with FAK inhibitor. The activation of FAK and the downstream AKT pathways was observed in MSCs cultured on the mesh-like scaffold. **d**, **e** Inhibitory effects of FAK and AKT on paracrine factor expression in MSCs cultured on the mesh-like scaffold detected via RT-PCR analysis and ELISA test. *p < 0.05, **p < 0.01, ***p < 0.001

It has been reported that the material topography, including surface pattern and roughness could affect the orientation and proliferation of cells via the material–cell interaction. These features also regulate various biochemical and biophysical signaling pathways, some of which modulate the paracrine function of MSCs [26]. In our study, we explore the underlying mechanism by which the mesh-like topography regulated the paracrine function of MSCs, and we focused on the FAK pathways, one of the most important pathways for ECM signal perception and mechanotransduction [27–29]. Activation of FAK triggers downstream PI3K pathway, with AKT serving as a key regulatory factor in stimulating cell survival, proliferation, differentiation, and promoting long-term cellular functionalities [30–32]. Therefore, we explored the expression of activated FAK and AKT of MSCs cultured on the mesh-like scaffold. Results showed that p-FAK, FAK, and the downstream p-AKT and AKT were significantly stimulated in BMSCs from the mesh-like scaffold group. To further validate the obtained results, the corresponding protein inhibitors were separately applied. After the co-incubation of the FAK inhibitor PF573228 with MSCs cultured on the PCL/M scaffold, the protein expression of p-FAK, FAK, and p-AKT was significantly downregulated in comparison with the control (Fig. 6c). Following the addition of the AKT inhibitor LY294002, the expression of p-AKT and AKT was effectively suppressed but with no effect on p-FAK expression (Additional file 1: Fig. S1). These observations indicated that FAK activation is upstream of AKT pathway. These inhibition studies confirmed that the phosphorylation of FAK was related to the activation of the downstream AKT pathways. In addition, to clarify the relationship between the FAK signaling pathway and the enhanced proangiogenic properties of BMSCs by mesh-like scaffold, we examined the expression levels of major proangiogenic factors produced by MSCs after the inhibition of FAK and AKT. The expression levels of typical angiogenic factors (VEGF, b-fgf) were substantially downregulated when FAK and AKT were inhibited (Fig. 6d, e). These results suggest that activation of FAK and the downstream AKT signaling might constitute the pathways required to regulate the paracrine function of MSCs by mesh-like topography (Fig. 6).

### Animal model of wound healing

**Fig. 7:**
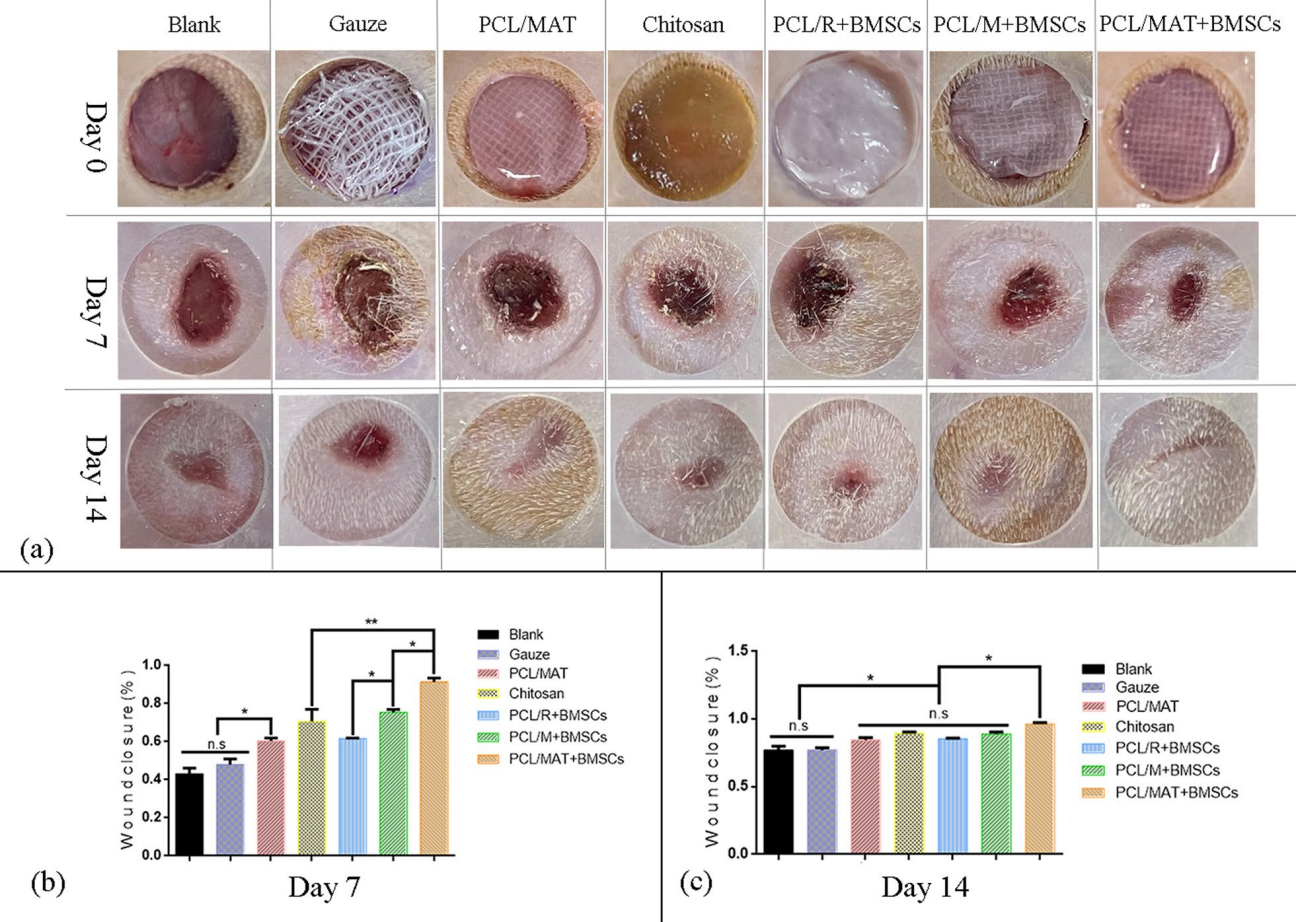
**a** Images on the day of surgery show that the initial wound covered with different dressings and the posterior images of the wounds at different time points (7 and 14 days) after surgery were sequentially arranged. **b**, **c** Statistics of the wound area at different time periods (7 and 14 days), which was used to characterize the wound healing of Blank, Gauze dressing, PCL/MAT, Chitosan dressing, PCL/R + BMSCs, PCL/M + BMSCs, and PCL/MAT + BMSCs dressing. *p < 0.05, **p < 0.01

To investigate the wound healing efficacy of PCL membranes seeded with BMSCs as artificial skin, a wound model was established by inflicting full-thickness skin wounds on the backs of rats. Figure 7 shows the images of the wounds on 0, 7, and 14 days after treatment with PCL/MAT + BMSCs, PCL/M + BMSCs, PCL/R + BMSCs, or chitosan dressing, acellular PCL/MAT membrane (PCL/MAT), gauze dressings, or blank. The negative control group had gauze dressing, while the positive control group had chitosan dressing. No membrane was applied to the blank control wounds. Acellular PCL/MAT membrane was used to examine the effects of ATV released from the scaffolds on wound healing. The wound areas were measured on days 7 and 14 to determine their healing. According to the image pixels (Fig. 7), the difference in healing rate was most significant in the first week, wounds shrank and fully closed after 14 d treatment. Wound healing was superior after PCL/MAT + BMSCs than after PCL/M + BMSCs treatment. Moreover, the latter accelerated healing more effectively than PCL/R + BMSCs. After 7 days treatment (Fig. 7b), the PCL/MAT + BMSCs, PCL/M + BMSCs, PCL/R + BMSCs, and chitosan dressing ratios were 91.41 ± 1.93%, 75.40 ± 1.54%, 61.66 ± 0.24%, and 70.60 ± 6.41%, respectively. It is worth noticing that the healing rate of PCL/MAT + BMSCs is significantly higher than the commercial chitosan dressing, indicating that PCL/MAT + BMSCs is an extremely effective dressing for reconstruction in wound sites, and could be considered a promising substitute for conventional dressing. The healing ratios of gauze dressings, blank, and PCL/MAT were 48.04 ± 2.84%, 43.13 ± 3.04%, and 60.44 ± 1.40%, respectively, in agreement with the previous studies of atorvastatin on wound healing [16].

### Histological analysis

**Fig. 8:**
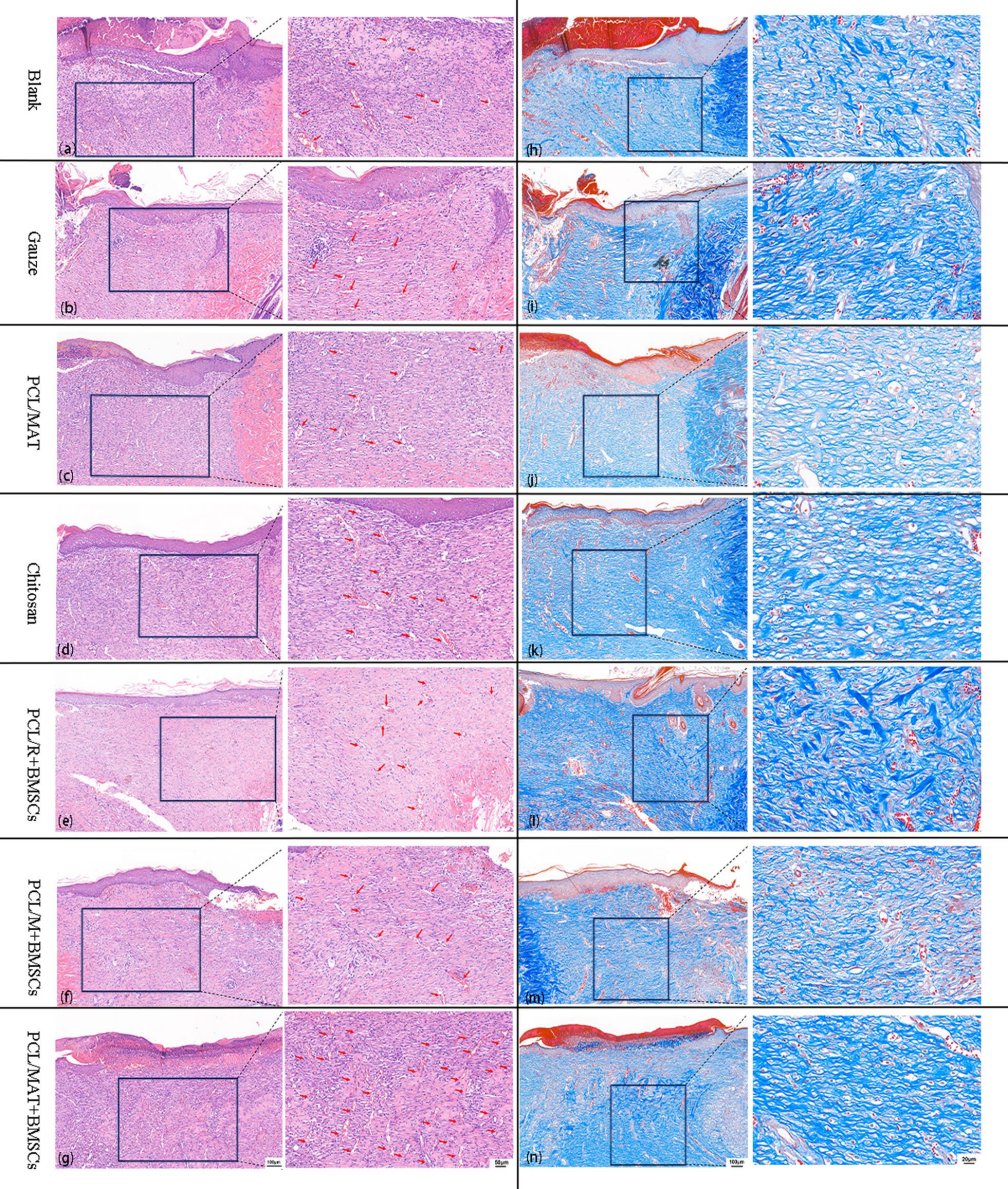
**a**–**g** H&E staining images of different groups of wound areas and detailed images of H&E staining corresponding to each group. **h**–**n** Masson's stained images of wound areas in each group and detailed images of Masson's staining corresponding to each group. The red arrow indicates the blood vessel. Blank, Gauze dressing, PCL/MAT, Chitosan dressing, PCL/R + BMSCs, PCL/M + BMSCs, and PCL/MAT + BMSCs dressing, respectively

**Fig. 9:**
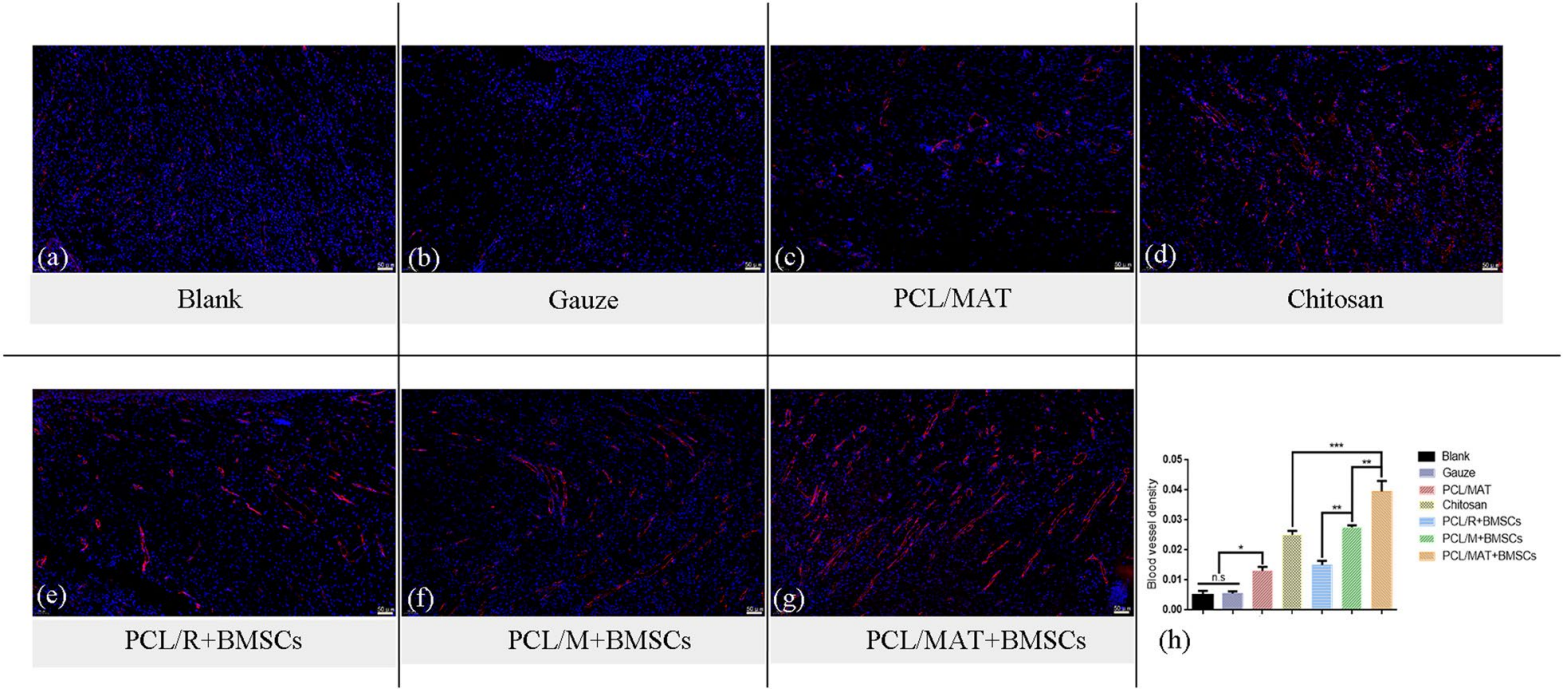
**a**–**g** Immunofluorescence staining and **h** quantification of CD31 detected in wounds to evaluate angiogenesis. *p < 0.05; **p < 0.01; ***p < 0.001. Blank, Gauze dressing, PCL/MAT, Chitosan dressing, PCL/R + BMSCs, PCL/M + BMSCs, and PCL/MAT + BMSCs dressing, respectively

H&E, Masson’s trichrome, and immunostaining were performed to evaluate wound healing histology. Seven days after wound induction, PCL/MAT + BMSCs induced significant wound healing, maximum angiogenesis, and compactly arranged collagen reconstruction compared with the other treatment groups (Fig. 8). The red arrow indicates the blood vessel in Fig. 8a–g. CD31 immunofluorescence staining revealed the blood vessel distribution in the wound tissue (Fig. 9). Figure 9g shows that vascularization was more abundant in the PCL/MAT + BMSCs than in the PCL/M + BMSCs. The vessels in the PCL/MAT + BMSCs group were arranged in parallel, and their orientation was perpendicular to the surface. Hence, this treatment group provided superior wound repair and granulation tissue formation. Moreover, the Masson’s trichrome staining images in Fig. 8h–n disclosed that the wounds treated with PCL/MAT + BMSCs were the densest and had neatly arranged collagen networks compared with the other groups. In PCL/M + BMSCs-treated group, the blood supply in the wound area was greater than that for PCL/R + BMSCs group (Fig. 9). Significantly, the blood vessel distribution of PCL/MAT + BMSCs group is much more abundant than that of the commercial chitosan dressing, again showing that the PCL/MAT + BMSCs is an extremely effective dressing for wound reconstruction, especially in promoting angiogenesis. The PCL/MAT-treated wounds had higher vessel density than those induced by the blank or gauze dressing groups. These results were consistent with previous studies of atorvastatin on wound healing [16].

M2 macrophages are essential for wound healing which could secrete high amounts of IL-10 and TGF-β to suppress inflammation, contribute to tissue repair, remodeling, vasculogenesis, and retain homeostasis [33]. It has been established that CD206 is classified into the marker of alternatively activated M2 phenotype and acts as a hemoglobin scavenger receptor on macrophage [34]. Thus, the wound repair condition can be reflected by counting the number of M2 macrophages through immunohistochemistry staining of CD206. As shown in Additional file 1: Fig. S2, in all groups, M2 macrophages were stained positively to various degrees. In particular, the density of the PCL/MAT + BMSC in the wound beds was the highest among all groups. Also, we found that CD206 M2-like cells were outnumbered in the mesh-like scaffold groups than in the random scaffold groups.

Thus, all of the histological results proved that the PCL/MAT seeded with BMSCs were conducive to epithelial regeneration, neovascularization, and anti-inflammatory effect to the greatest extent.

## Discussion

MSCs have been extensively investigated for tissue engineering due to their strong regenerative potential [35]. Studies have shown that paracrine effect of MSCs rather than their direct differentiation into mature tissue exerts the main therapeutic function in cell therapy. The MSCs respond differently to specific microenvironments in host tissue [9, 10]. Angiogenesis plays a vital role in tissue engineering and has been attracting attention [36, 37]. It is a key step in tissue regeneration as it is required to supply nutrients, oxygen, and circulating progenitor cells to injured tissues [36]. Thus, angiogenesis is being increasingly evaluated for assessing the trophic effect of MSCs.

Combinatorial use of cells and biomaterials is an important approach in cell-based therapies. Understanding the interactions between cells and biomaterials and their associated mechanisms is essential to develop functional materials for regenerative medicine [38]. The influences of various materials on cells have been previously reported. It may be possible to improve the paracrine capacity of MSCs by manipulating characteristics of biomaterials such as topography. This approach might improve the efficacy of MSC-based treatments since fibrous topography is reported to induce MSC extension and cytokine release [35, 39]. An earlier study designed and fabricated MSC-seeded scaffold as tissue-engineered skin with high biological activity and mechanical properties resembling those of natural skin. This construct strongly induced cell migration and upregulation of genes expressing associated healing factors, showing excellent wound healing characteristics [40].

To the best of our knowledge, it is the first time to evaluate the impact of a combination of mesh-like topography and atorvastatin loading on the paracrine function of MSCs. Our study showed that the paracrine function of BMSCs seeded on atorvastatin-loaded mesh-like scaffold (PCL/MAT) was markedly greater than that for BMSCs seeded on a random scaffold (PCL/R) or a drug-free mesh-like scaffold (PCL/M). The PCL/MAT scaffold creates a conducive microenvironment for the BMSCs seeded upon it. The mesh-like topography of fibrous scaffolds upregulated the secretion and gene expression of proangiogenic cytokines in BMSCs, and the atorvastatin released from these scaffolds augmented this response (Fig. 5f, g). Hence, a combination of the two enhanced the angiogenic effect of the BMSCs seeded on the membrane. In our study, BMSC-seeded PCL/MAT membrane used as tissue-engineered skin in an *in vivo* wound healing model substantially promoted angiogenesis and collagen reconstruction.

Fibers may be arrayed by electrospinning technology into mesh-like or random phenotypes with distinct and specific surface morphologies and mechanical properties [41]. Previous studies showed that certain surface topographies had superior tissue regeneration potential because their structures provided topographic cues to adherent cells [35, 41, 42]. FITC-DAPI staining (Fig. 4b–d) showed that compared with BMSCs cultured on random mats, those grown on mesh-like membranes were relatively elongated and formed new cytoskeletons by extending their pseudopodia, leading to diverse morphologies. These results confirmed that the growth of BMSCs may be induced by mesh-like topographies. Next, we explored the potential mechanism by which the mesh-like topography modulated the paracrine profiles of MSCs and found out that MSCs cultured on mesh-like scaffold were with higher FAK activation than on random scaffold (Fig. 6). We found that mesh-like topography regulated the secretion of angiogenic factors of BMSCs via activation of FAK and the downstream AKT signaling pathway. Similarly, FAK signaling has been reported to mediate multiple cellular responses toward the physical cues provided by biomaterials [43–45]. Interestingly, previous studies have shown that focal adhesion kinase (FAK), downstream AKT signaling might participate in the required mechanotransductive pathways, through which the structures stimulated the paracrine function of MSCs [46]. Moreover, it has been reported that the mechanical effect could enhance the expression of adhesion molecule (FAK) and significantly increase the release of the factors VEGF and b-FGF from stem cells [47]. Meanwhile, other studies revealed that PI3K/AKT pathways might participate in the mechanical effect on the biological characteristics of BMSCs [48]. Therefore, it is reasonable to speculate that FAK/AKT signaling might be involved in mediating the secretion of angiogenic factors of MSCs by mesh-like scaffold. Consistent with previous studies, we found that FAK and p-FAK in BMSCs were significantly elevated on PCL/M (Fig. 6). The involvement of FAK signaling in regulating the angiogenic properties of BMSCs was further confirmed using the FAK inhibitor PF573228 (Fig. 6). Chemical signals from the membrane provide vital cues for BMSC paracrine secretion. Here, we selected ATV to enhance the activity of mesh-like PCL membranes because this drug promotes wound healing [16]. Orally administered statins induce multiple side effects including myopathy and hepatotoxicity [49]. Thus, topical statin delivery is a promising route of drug administration for wound healing as it provides more effective drug delivery, leads to prolonged action, and leads to fewer side effects [50]. Using a simple, efficient mixture electrospinning technology, we incorporated ATV into the mesh-like PCL membrane. FTIR analyses demonstrated successful ATV loading (Fig. 2d). The release of ATV is regulated by the pores in and on the fibers. This sustained-release system could deliver ATV for at least 1 week. ATV protects MSCs against harsh microenvironments and their deleterious effects such as hypoxia and serum-free injury by inducing AMP-activated protein kinase [19]. It was reported that ATV improves MSC transplantation efficacy after acute myocardial infarction and therapeutic MSC implantation in acute kidney injury [51–53]. In earlier studies, VEGF mRNA and protein levels were upregulated in MSCs cultured on statin-loaded scaffolds [21], while exosomes derived from ATV-pretreated MSCs accelerated diabetic wound repair by enhancing angiogenesis via the AKT/eNOS pathway [18]. In the present study, b-FGF and VEGF gene expression and protein secretion were relatively increased in BMSCs seeded on drug-loaded mesh-like membranes (Fig. 5f, g). Moreover, the effects of the BMSC CM on tube formation and endothelial migration possibly reflect the influences (Fig. 5b–e). Hence, sustained ATV release in the drug-loaded system and the mesh-like characteristics may increase the gene expression and protein secretion of VEGF and b-FGF. To rule out the potential effects of ATV release on HUVEC migration and proangiogenic function, we used the leachate solution from ATV-loaded membranes to culture the cells. The released ATV increased the total length of the vessel-like tubes and accelerated HUVEC migration. These findings were consistent with our in vivo animal experiment results.

Protection from the harmful effects of the external environment and establishment of stable vascular repair conditions at the wound site are of vital importance. An ideal biological wound tissue engineering barrier is biocompatible and conducive to cell adhesion and proliferation [54, 55]. It must also be mechanically strong and possess chemical characteristics that support effective wound healing [5, 14]. Here, PCL was fabricated as a mesh-like membrane loaded with ATV and seeded with BMSCs. Similar to other electrospun scaffolds [56, 57], our PCL membranes had high specific surface area, aspect ratio, and microporosity [54]. Hence, their architecture resembled those of natural ECM and skin. Moreover, they delivered cytokines and ATV to the wound area and exhibited excellent tensile strength (Fig. 2f) [56]. As shown in the mechanical tests, the random membrane showed higher Young’s modulus and tensile stress than the mesh-like membrane (Fig. 2f–h). Hence, the special structure makes mesh-like membrane more elastic and flexible than random membrane.

**Fig. 10:**
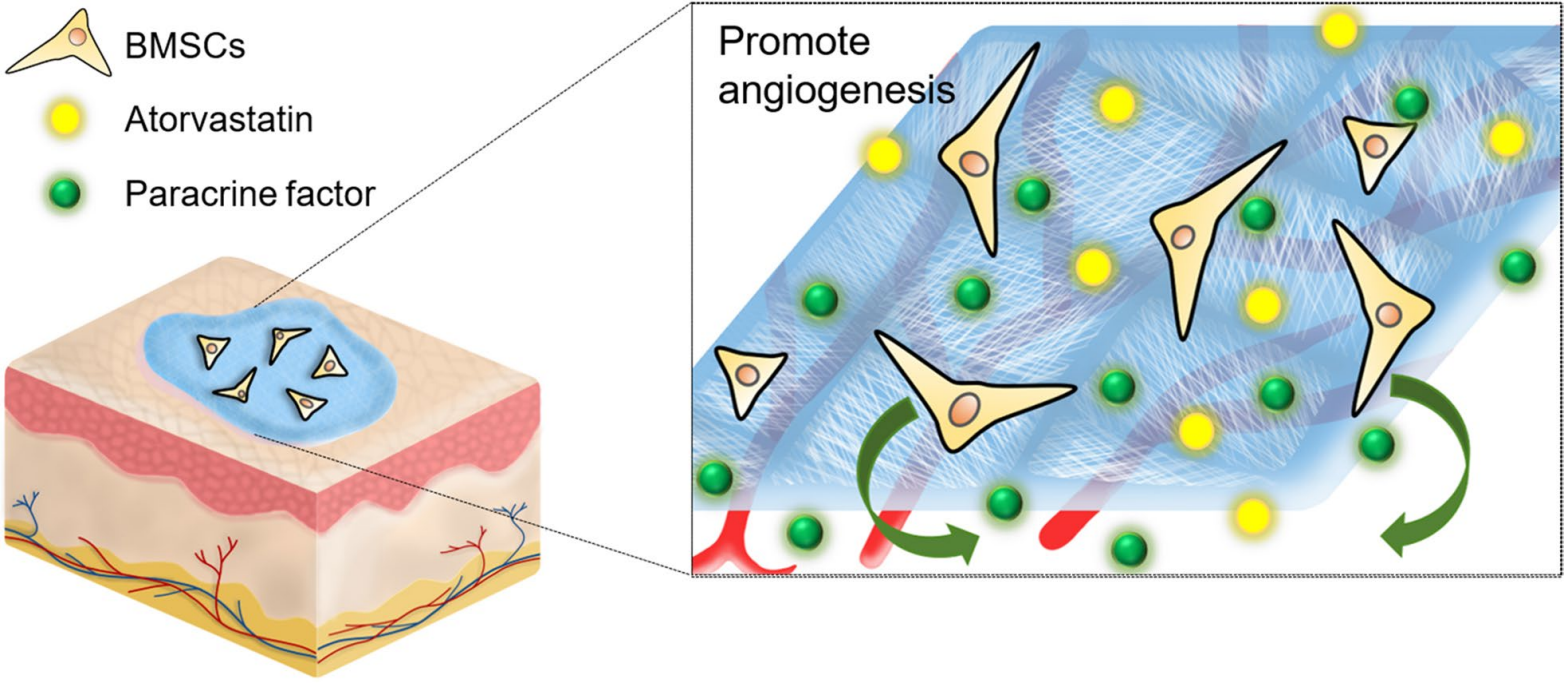
Schematic diagram illustrating the effect of mesh-like structure signals combined with atorvastatin chemical signals on the paracrine action of BMSCs and the PCL/MAT-BMSCs membrane works as barrier protection on wound area

The suitable fiber microstructure and excellent biocompatibility of our membranes improved cell adhesion, migration, and proliferation which are critical for a wound barrier. Electrospinning research over the past decade has confirmed the biosafety of PCL [58–60]. The in vitro test results in Fig. 4a demonstrated no significant cytotoxicity for PCL/R, PCL/M, or PCL/MAT (ATV = 2.5 mg). The DAPI-FITC staining images in Fig. 4b–d revealed that BMSCs cultured on membranes have normal morphology including cellular antennae and pseudopodia. These cells successfully adhered and proliferated on all test surfaces. Normal BMSC adhesion and growth are vital for paracrine secretion and wound healing [40, 41]. We established a rat model of wound healing to determine the influences of PCL/MAT + BMSCs on wound areas. The wound area of the PCL/MAT + BMSCs group was significantly smaller than those of the other groups at each time point (7 and 14 days) (Fig. 7), even better than the commercial chitosan dressing. Histological analyses by H&E, Masson’s trichrome, and immunostaining disclosed that the rat wounds covered with the PCL/MAT + BMSCs presented the highest degree of angiogenesis, the densest collagen reconstruction, and the highest density of M2 macrophages. These results confirmed that suitable microstructure, excellent biosafety, and effective biochemical factors make PCL/MAT + BMSCs dressing highly dependable as a protective barrier for epithelial regeneration, neovascularization, and anti-inflammatory. Therefore, the PCL/MAT + BMSCs membrane is reliable as an artificial skin and has excellent wound healing properties and could be considered a promising substitute for conventional dressing (Fig. 10).

## Conclusion

Mesh-like topography combined with atorvastatin load cooperates to create a unique microenvironment that upregulates the expression of proangiogenic cytokines in BMSCs. This configuration demonstrated therapeutic efficacy in both in vitro assays and a full-thickness in vivo wound healing model. More importantly, the paracrine function of MSCs cultured on the mesh-like scaffold was improved via FAK and the downstream AKT pathways to upregulate the secretion of angiogenic factors, suggestive of the potential presence of topographical-dependent MSC secretion. This is a novel design clarified cell–material interactions wherein the topographical properties of the biomaterial and the mode of action of its drug load were integrated to establish cell signaling and communication networks that promote the paracrine functions of BMSCs and tissue regeneration. It is essential for the development of functional materials for future regenerative medicine and could be a promising approach to solve the traumatic skin defect and accelerate recovery.

## Supplementary Information

**Additional file 1.** Semi-quantitative analysis of FAK and AKT signaling pathway with AKT inhibitor and immunohistochemistry staining of CD206 for M2 macrophages analysis.

## Data Availability

The datasets used and/or analyzed during the current study are available from the corresponding author on reasonable request.

## Abbreviations

BMSCs: Bone marrow stem cells
HUVECs: Human umbilical vein endothelial cells
ATV: Atorvastatin
CM: Conditioned medium
EFs: Electrospun fibers
MP: Microplate
PCL/R: Polycaprolactone random electrospun fibrous
PCL/M: Polycaprolactone mesh-like electrospun fibrous
PCL/MAT: Polycaprolactone mesh-like membranes loaded with atorvastatin
RT-PCR: Real-time polymerase chain reaction
VEGF: Vascular endothelial growth factor
b-FGF: Basic fibroblast growth factor
