# Supplementary figures and images for: Mesh-like electrospun membrane loaded with atorvastatin facilitates cutaneous wound healing by promoting the paracrine function of mesenchymal stem cells
Source: Stem Cell Res Ther. 2022 May 7;13:190. doi: 10.1186/s13287-022-02865-5 (PMC9080129; doi:10.1186/s13287-022-02865-5)

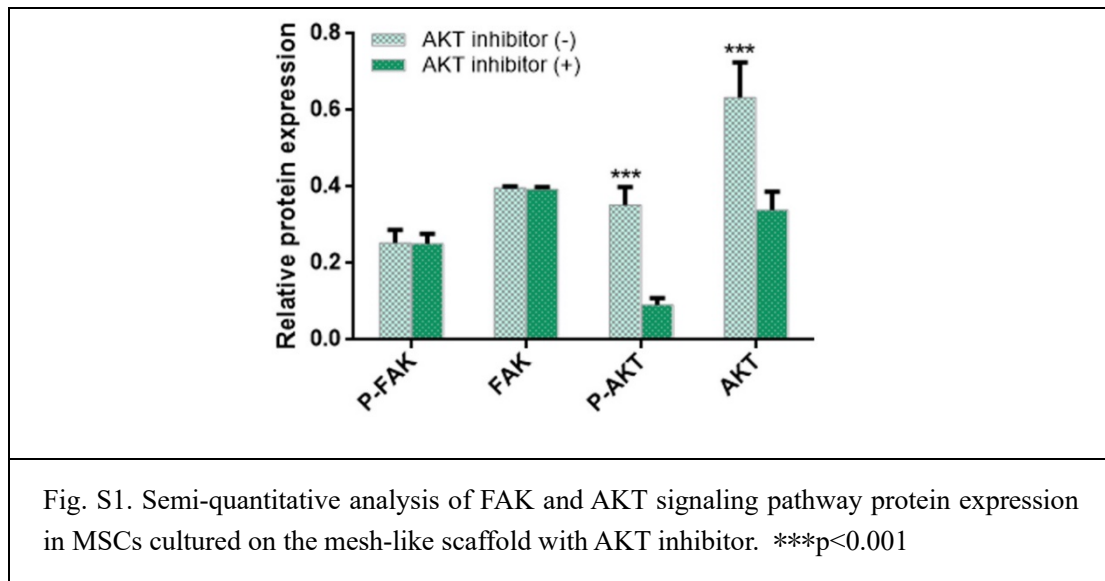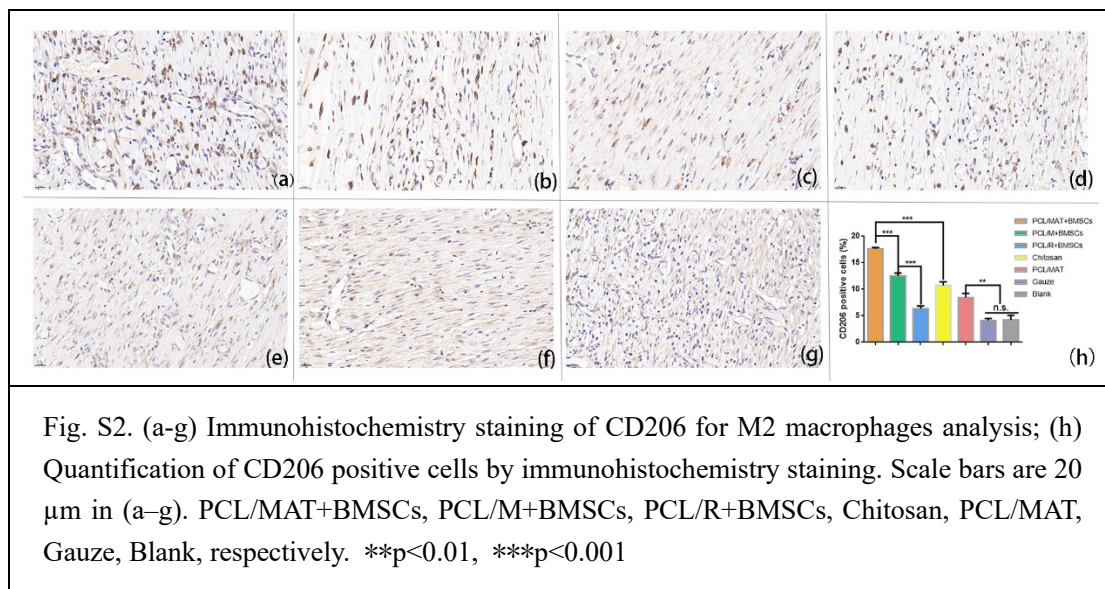

Supplement: Supplementary file 1 — Additional file 1. Semi-quantitative analysis of FAK and AKT signaling pathway with AKT inhibitor and immunohistochemistry staining of CD206 for M2 macrophages analysis. [file 13287_2022_2865_MOESM1_ESM.pdf]
